# Efficiency Enhancement of InGaN-Based Solar Cells *via* Stacking Layers of Light-Harvesting Nanospheres

**DOI:** 10.1038/srep28671

**Published:** 2016-06-24

**Authors:** Amal M. Al-Amri, Po-Han Fu, Kun-Yu Lai, Hsin-Ping Wang, Lain-Jong Li, Jr-Hau He

**Affiliations:** 1 King Abdullah University of Science & Technology (KAUST), Computer, Electrical and Mathematical Sciences and Engineering Division, Thuwal, 23955-6900, Saudi Arabia; 2 National Central University, Department of Optics and Photonics, Chung-Li 32001, Taiwan; 3 King Abdullah University of Science & Technology (KAUST), Physical Science and Engineering Division, Thuwal, 23955-6900, Saudi Arabia

## Abstract

An effective light-harvesting scheme for InGaN-based multiple quantum well solar cells is demonstrated using stacking layers of polystyrene nanospheres. Light-harvesting efficiencies on the solar cells covered with varied stacks of nanospheres are evaluated through numerical and experimental methods. The numerical simulation reveals that nanospheres with 3 stacking layers exhibit the most improved optical absorption and haze ratio as compared to those obtained by monolayer nanospheres. The experimental demonstration, agreeing with the theoretical analyses, shows that the application of 3-layer nanospheres improves the conversion efficiency of the solar cell by ~31%.

InGaN-based photovoltaic (PV) devices have recently drawn much research attention because of the large overlap between the alloy’s bandgap and the solar spectrum^1^,^2^,^3^. In addition to the known favorable PV properties (e.g. high absorption coefficient and high carrier mobility)^4^,^5^, it is recently found that the solar cell, with InGaN/GaN multiple quantum wells (MQWs) as the active region, exhibits superior resistance in high-temperature and high-energy radiation conditions, showing the potential for harsh environment applications^6^. Despite these promising properties, the performances of InGaN-based solar cells still require much improvement as most of the reported conversion efficiencies (η) are not commercially competitive^2^,^3^,^6^,^7^. The improvement can be achieved via advanced growth techniques to attain superior crystal qualities^8^,^9^. Alternatively, η can be enhanced by fabricating antireflective (AR) surface structure to increase optical transmission through the air/device interface^10^,^11^.

Among the many published methods for AR surface fabrication, nanosphere lithography is a popular one in light of its simplicity and capability to yield various geometrical features^11^,^12^,^13^,^14^,^15^. The technique employs a monolayer of self-assembly colloidal nanospheres as the etching mask or the intermediate medium providing impedance match between air and device surfaces^15^. There has been much efforts devoted to optimize the performances and effective area of nanosphere-based AR coating^12^,^13^,^16^. However, it is shown that attaining a monolayer of nanospheres with large-area uniformity is rather difficult, which is due to the limited control over the kinetics of particles on wafer surfaces. One way to increase the effective area of AR nanospheres is to increase the number of stacking layers and allow a certain irregularity in the nanosphere assembly. In comparison with the monolayer structure, the nanospheres with multiple stacking layers are more likely to yield large-area coverage without sacrificing AR performances. More importantly, stacking the nanospheres has been demonstrated as an effective approach to enhance Mie scattering in a wide range of wavelengths^17^, which is strongly desired for solar cells considering the light trapping effect induced on device surface^18^. Although it is easier to realize the AR coating of improved effective area and scattering property with stacking layers of nanospheres, the application of stacking nanospheres on photovoltaic devices are scarcely reported.

In this study, the effect of stacking polystyrene (PS) nanospheres on the performances of InGaN-based solar cells is systematically investigated. Theoretical analyses based on finite-difference time-domain (FDTD) and rigorous coupled-wave analysis (RCWA) methods indicate that the AR performance of nanospheres is enhanced when the number of stacking layers increases from 1 to 3. The stack exceeding 3 layers of PS nanospheres leads to lower absorption efficiencies of PV devices because of the decreased optical transmission through nanospheres. The simulation result agrees with the experimental observation that photocurrents of the InGaN/GaN MQW solar cell is noticeably increased upon the application of stacking PS nanospheres (1–3 layers), leading to the η enhancement of ~31%. The concept presented here should benefit the development of InGaN solar cells, as well as the light-harvesting scheme applicable to a wide variety of optoelectronic devices.

## Results

**Figure 1:**
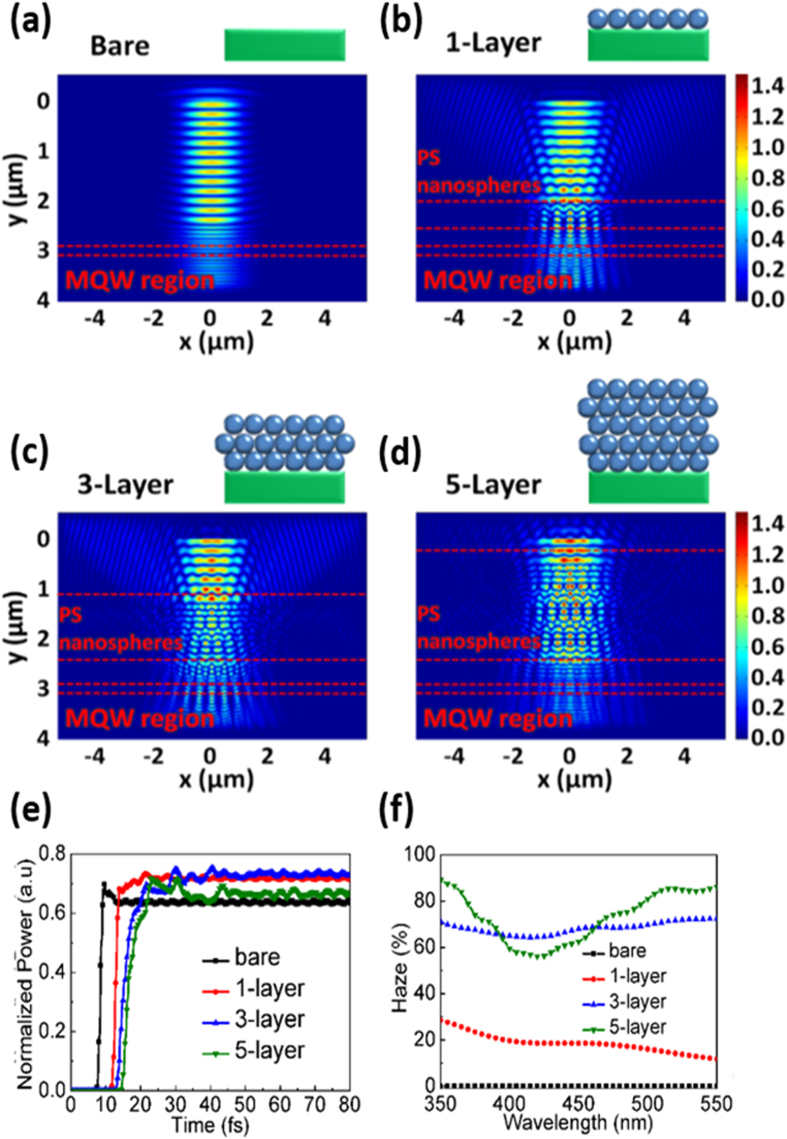
FDTD simulation of TE electric field distribution, |Ez|, within the solar cells of different surface conditions: (**a**) bare; (**b**) 1-layer; (**c**) 3-layer; and (**d**) 5-layer of nanospheres. The regions of MQWs and PS nanospheres are indicated by red dash lines. (**e**) Normalized optical power, integrated over the MQW region, as a function of time. (**f**) Transmission haze ratio within the device simulated by RCWA analysis.

The dimension of PS nanospheres used in this study is 450 nm in diameter, which is commonly adopted by many groups involved in self-assembly nanoengineering. In order to determine the optimized stacking number of nanospheres rendering the largest optical power transmitted to the quantum wells, numerical calculations based on FDTD and RCWA were performed. Figure 1(a–d) show time-averaged TE-polarized electric field intensity, |E_z_|, distribution within the solar cells covered by 0-, 1-, 3- and 5-layer nanospheres, respectively. Schematics of the relative position of each stacking nanospheres are also shown in the figures. Compared to the device of bare surface, the field intensities in the MQW region are enhanced with PS nanospheres. The nanospheres not only facilitate light propagating across the interfaces by blurring the abrupt index transition from air to ITO layer, but also broaden the field distribution within the device because of the increased scattering effect, which is expected to increase in the absorption possibility in active regions. The strong field intensities in the nanosphere region indicate that the nanospheres act as effective scattering centers, giving rise to pronounced light-harvesting effect. Figure 1(e) presents the normalized optical power, integrating the power flux over the MQW regions, as a function of time. The steady-state integrated power values are 0.63, 0.72, 0.74, and 0.66 for the nanospheres with 0, 1, 3, 5 stacks, respectively. The results indicate that properly increasing the stacking number of nanospheres leads to the maximized enhancement of optical absorption by the solar cell. In order to further investigate the scattering effect induced by the stacking nanospheres, we adopt RCWA algorithm to calculate the transmission haze ratios at the absorption wavelengths of the MQWs. The haze ratio is defined as:


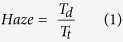


where T_d_ is the diffused transmittance representing the multiple order scattering and T_t_ is total transmittance. Figure 1(f) shows the haze ratios with the stacking number of 0, 1, 3, 5. Compared to the monolayer nanospheres, the high haze ratios are dramatically enhanced with increased stacks of nanospheres, which can be explained by the scattering tendency of light impinging on the nanospheres. The scattering modes introduce additional lateral components in the forward wave vectors, leading to increased optical paths on device surfaces and thus the high haze ratios. However, one can see that the haze ratios of the 5-layer nanospheres become less than those of the 3-layer stack at the wavelengths of 390–460 nm. The optical power transmitted to the MQW through the 5-layer nanospheres is also found to be decreased as compared with the case of 3-layer ones [Fig. 1(e)]. The results can be attributed to the feature of Mie scattering [applicable when the wavelength (λ) is comparable to the nanosphere diameter (d)]. In general, the intensity of Mie scattering is higher in the forward direction than in the reverse direction, and the forward intensity becomes stronger with larger d/λ ratios^19^. In other words, the decrease of haze starting from λ = 350 nm is due to the weaker forward scattering with smaller d/λ values. Since the 5-layered sample contains more nanospheres than the 3-layered one, stronger wavelength dependence is observed and the weaker forward scattering can also lead to excessive light trapped in the nanospheres, resulting in the decreased absorption by MQW. As the wavelength increases beyond 500 nm, the nanospheres become less resolved and the increased haze should be explained with the effective medium theory, in which the grading of refractive index promotes optical transmission through the interface^10^,^12^.

**Figure 2:**
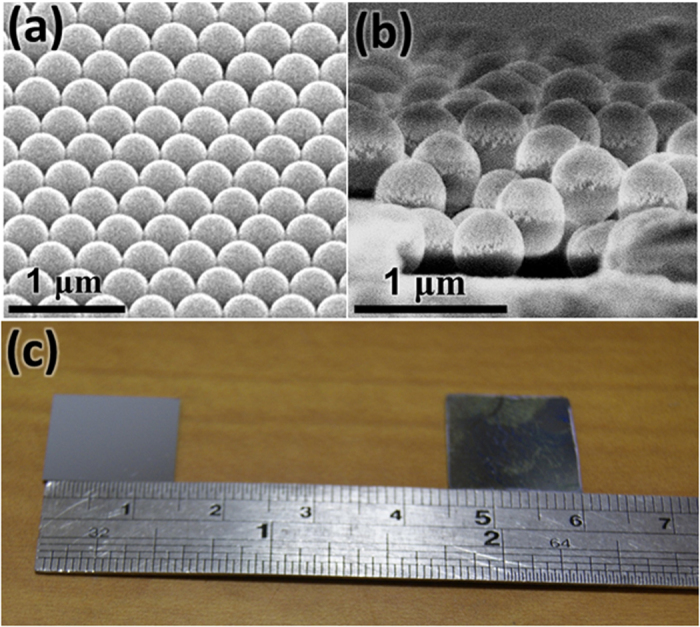
SEM images of (**a**) the monolayer and (**b**) the multilayers of PS nanospheres. The nanospheres are coated with Au to avoid electron charging for SEM observation. (**c**) Photographs of the bare Si wafer surface and the one covered with multilayered nanospheres.

Figure 2(a,b) respectively present the scanning electron microscopy (SEM) image of the monolayer and the multiplayers of the nanospheres on the MQW solar cells. In Fig. 2(b), one can estimate the height of stacked nanospheres is in the range of 1–1.5 μm, being close to the total layer number of 3 considering the nanosphere diameter (450 nm) and the hexagonal close-packed (HCP) stacking arrangement. It should be mentioned that precisely controlling the thickness of multilayered PS nanospheres is rather difficult since nanospheres tend to partially fall from the rigid HCP positions, and the variation in stacking height is expected to be increased with larger layer numbers. Nevertheless, we would like to emphasize that allowing a certain height variation while maintaining sufficient haze ratio is the key to reach large effective area of the antireflective nanospheres. Figure 1(c) shows the photograph of a 1.5 × 1.5-cm^2^ bare silicon wafer surface and the one covered with multilayered nanospheres. The difference in surface reflection between the two samples can be easily perceived with naked eyes. The monolayered sample is not shown in the photograph because of very limited uniform area (<0.1 × 0.1 cm^2^).

**Figure 3:**
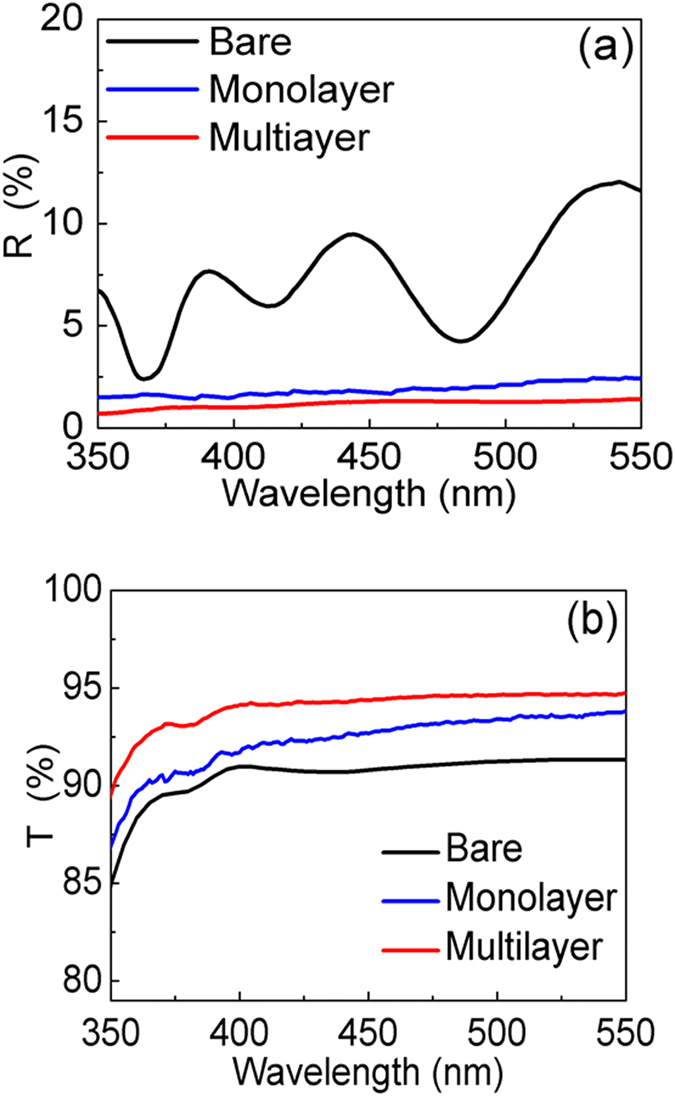
Specular (**a**) reflectance and (**b**) transmittance measured with bare surface, monolayer and multilayer of PS nanospheres.

**Figure 4:**
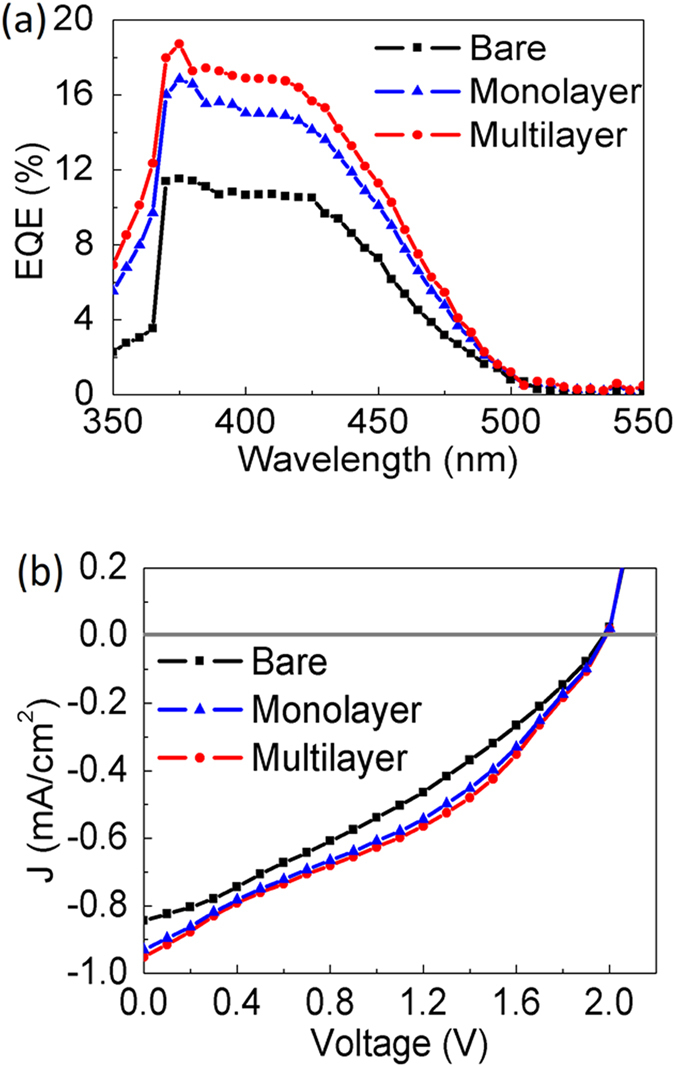
(**a**) EQEs (**b**) J-V characteristics measured on the MQW solar cells with bare surface, monolayer and multilayer of PS nanospheres.

Figure 3(a) shows the reflectance spectra measured on the MQW solar cells with bare surface, monolayer and multilayer of PS nanospheres. The transmittance spectra recorded on glass slides with the three different surface conditions are presented in Fig. 3(b). As expected, the highest transmittance is attained with the multilayered sample, followed by the monolayered, and the bare glass allows the least transmitted light. The consistency between the reflectance and transmittance spectra indicates that the scattering effect induced by stacked nanospheres can facilitate optical waves passing through the interfaces, instead of bouncing the light back to air. In Fig. 3(a), the oscillation observed on the bare surface is owing to the interferences occurring at the layer interfaces. The reflectance minimum at the wavelength of 365 nm arises from the bandgap absorption of GaN. The results demonstrate that the nanospheres are effective in suppressing the undesired Fresnel reflections over a wide range of wavelengths. The significantly lower reflection on the nanospheres can be ascribed to several effects. Firstly, the refractive index n of polystyrene is around 1.59 at λ = 500 nm^13^, mitigating the abrupt index transition from air (n = 1) to ITO (n = 2.04 at λ = 500 nm)^14^. The graded index transition allows enhanced optical transmission into the semiconductor. Secondly, since the size of the nanospheres is comparable to the incident wavelengths, intensive scattering can be induced among the nanospheres, giving rise to the light harvesting effect^18^. As predicted in Fig. 1(f), the multilayer nanospheres are expected to exhibit the strongest scattering effect and display the lowest reflectance among the three samples. The effects of index grading on the light harvesting property would boost the optical absorption in the MQW region, resulting in enhanced photovoltaic performances. Figure 4(a) presents the external quantum efficiency (EQE) spectra of the devices with bare surface, monolayer, and multilayer of nanospheres. The spectra were recorded under monochromatic illumination by a halogen lamp coupled to a monochromator. One can see the EQEs are much enhanced after the application of nanospheres. It is also noticed that the EQE peak of the MQW solar cell does not shift upon the application of the nanospheres, indicating that the absorption behavior of the device is not affected by the nanospheres. The boosted EQEs of the nanosphere-covered devices mainly come from the increased absorption in the MQW, as mentioned in the discussion of Fig. 1(e). Since the stacking number of the multilayer structure is close to 3, with which the haze ratio is much larger than that of the monolayer structure, the solar cell coated with multilayer nanospheres delivers the highest EQEs. As the Mie scattering efficiencies increase at shorter incident wavelengths^19^,^20^, one can observe that the EQE enhancement brought by the nanospheres becomes increasingly pronounced at the wavelengths below 430 nm. It is worthwhile to mention that the range of spectra response seen in Fig. 4(a) extends to the wavelengths beyond 500 nm, which is wider than many published results^6^,^7^,^21^. The broad EQE spectra are usually observed with the MQW with high indium contents^3^,^22^, which tend to exhibit indium segregation and thus result in inhomogeneous distribution of bandgap energies^2^.

Figure 4(b) displays the current density-voltage (J-V) curves of the solar cells measured with a Keithley 4200 source meter under the illumination of 1 sun air mass 1.5 global solar simulator. The photovoltaic characteristics are summarized in Table 1. In comparison to the bare device, the multilayer of nanospheres lead to the increased short-circuit current density (Jsc), echoing the result seen in the EQE spectra. Although the Jsc enhancement is limited, which can be due to the decreased irradiance of solar spectrum at the wavelengths below 450 nm, the multilayer nanospheres improve the η to be 0.59%, which is ~31% higher than that of the bare device. The relatively low fill factors, being closely related to η, should be caused by the crystal imperfections in high-indium-content MQWs^3^. Photovoltaic performances of the MQW solar cell can be improved by optimizing the layer structure, such as the number of quantum wells and surface roughness of the device^21^.

## Conclusion

Light-harvesting scheme with multilayer PS nanospheres on InGaN-based MQW solar cells is demonstrated via theoretical and experimental methods. It is found that the effective area and the light-harvesting performances can be enhanced by controlling the stacks of nanospheres within 1–3 layers. The InGaN/GaN MQW solar cell covered with the multiplayer nanospheres exhibits around 31% enhancement of η without perturbing the absorption behavior of the device. The improved η is mainly attributed to the enhanced forward light scattering induced among the nanospheres, which is analyzed with FDTD and RCWA calculations. The concept and fabrication scheme presented here open a new path for tailoring light into PV devices.

## Methods

### Solar Cell Fabrication

The MQW solar cells were grown by metal-organic chemical vapor deposition on c-plane sapphire substrates. The layer structures consist of nine periods of intentionally undoped In_0.3_Ga_0.7_N (3 nm)/GaN (17 nm) MQWs, sandwiched by a 2.5-μm n-type and a 0.2-μm p-type GaN layer. In device fabrication, indium tin oxide (ITO) was deposited by electron beam evaporation on p-GaN to form transparent Ohmic contacts. The 1 × 1 mm^2^ diode mesas were then defined by chlorine-based plasma etching. The contacting scheme consists of interdigitated Ti/Al/Ni/Au metal grids deposited on the ITO and n-GaN. The PS nanospheres are 450 nm in diameter. In the coating process, the nanospheres were firstly diluted in deionized water and applied to the device surface by drop coating. The device was then held in an incubator at the temperature of 50 °C and 87.5% of humidity for 2 hours. Layer thickness of nanospheres was tuned by the tilt angle of the sample during drying in the incubator. The multilayered nanospheres were formed at the tilt angle less than 5°, while the monolayer sample was attained at around 15°.

### FDTD Analysis

The analysis was carried out to reveal the propagation behavior of incident light across the nanosphere/device interface. Steady-state electromagnetic field distributions are calculated by selecting proper refractive indices (n and k) for all materials^23^,^24^,^25^. The grid size is Δx × Δy = 0.01 × 0.02 μm^2^ in the space domain, and the time interval between every calculation is 0.027 fs. Boundaries in x and y directions are surrounded by the 0.5 μm perfectly matched layers to absorb the evanescent electromagnetic waves^24^. The excitation source (placed at y = 0) is a continuous Gaussian beam of 2 μm in width. The wavelength for all simulation is selected to be 375 nm, at which the peak of EQE is observed. All of the calculated values are normalized to the ones of the excitation source.

## Additional Information

**How to cite this article**: Al-Amri, A. M. *et al*. Efficiency Enhancement of InGaN-Based Solar Cells *via* Stacking Layers of Light-Harvesting Nanospheres. *Sci. Rep.* **6**, 28671; doi: 10.1038/srep28671 (2016).

## Figures and Tables

**Table 1:** Photovoltaic characteristics of the MQW solar cells with different surface conditions.

Surface Condition	Bare	Monolayer	Multilayer
Voc (V)	1.9	1.9	1.9
Jsc (mA/cm^2^)	0.85	0.93	0.95
Fill Factor (%)	27.58	32.03	32.74
η (%)	0.45	0.57	0.59
